# Supplementary material for: The spatiotemporal richness of hummingbird wing deformations
Source: J Exp Biol. 2024 May 21;227(10):jeb246223. doi: 10.1242/jeb.246223 (PMC11166462; doi:10.1242/jeb.246223)
Supplement: Supplementary information [file jexbio-227-246223-s1.pdf]

Table S1. Average morphological measurements of the five individual hummingbirds in this study. Values in flight are presented as means and 95% confidence level. For individuals 4 and 5, we also report measurements from posed specimens based on the wing's silhouette or the landmarks on the wing perimeter, together with the percentage difference from the in-flight measurement. Non-dimensional second moment of area, $\hat{r}_2(S)$.

**Bird**	**Body mass (g)**	**Length (cm)**	**Area (cm^2)**	$\hat{r}_2(S)$
1	4.08 (0.03)	4.969 (0.008)	6.60 (0.02)	0.503 (0.003)
2	3.78 (0.01)	5.128 (0.002)	6.60 (0.01)	0.497 (0.001)
3	3.93 (0.04)	4.909 (0.006)	6.05 (0.02)	0.492 (0.001)
4	4.11 (0.04)	4.972 (0.004)	6.34 (0.03)	0.501 (0.001)
4 (posed silhouette)		4.855 (-2.4%)	6.85 (+10%)	0.491 (-2.1%)
4 (posed markers)		4.733 (-4.8%)	6.12 (-3.5%)	0.483 (-3.6%)
5	4.18 (0.02)	5.027 (0.007)	6.35 (0.03)	0.487 (0.001)
5 (posed silhouette)		4.633 (-7.8%)	6.17 (-2.8%)	0.498 (+2.2%)
5 (posed markers)		4.552 (-9.5%)	5.53 (-13.0%)	0.481 (-1.3%)

Journal of Experimental Biology • Supplementary information

Table S2. Kinematic and morphological variation among hummingbirds during identified phases of three flight conditions. Values are presented as means and 95% confidence level for the number of individuals (N) in each condition. Midspan section angle relative to vertical, α. Non-dimensional second moment of area, $\hat{r}_2(S)$.

Stroke phase	Condition	N	Position (°)	Elevation (°)	α (°)	Length (cm)	Area (cm^2)	$\hat{r}_2(S)$	Twist (°)	Chordwise camber (%)	Spanwise camber (%)
Pronation	Visual mask	4	49.12 (5.697)	19.347 (3.854)	76.422 (2.846)	4.722 (0.04)	5.455 (0.147)	0.487 (0.004)	4.58 (3.597)	5.248 (0.718)	6.455 (0.637)
	Typical flight	5	28.388 (7.415)	22.239 (2.392)	83.548 (1.917)	4.749 (0.048)	5.47 (0.12)	0.485 (0.003)	7.472 (2.781)	4.321 (0.731)	6.954 (0.719)
	Submaximum load lifting	3	24.881 (10.332)	23.123 (2.031)	84.26 (1.834)	4.752 (0.012)	5.452 (0.069)	0.485 (0.003)	6.485 (3.534)	5.18 (0.62)	7.123 (1.096)
Mid-downstroke	Visual mask		93.185 (5.5)	9.439 (5.566)	29.593 (3.014)	4.96 (0.062)	6.414 (0.188)	0.499 (0.003)	-36.98 (2.656)	10.048 (0.447)	7.391 (0.697)
	Typical flight		91.276 (5.475)	7.392 (2.318)	20.719 (2.275)	5.001 (0.039)	6.388 (0.106)	0.496 (0.003)	-35.456 (1.665)	12.033 (0.43)	6.033 (0.565)
	Submaximum load lifting		98.195 (8.605)	12.79 (1.261)	19.866 (3.813)	5.031 (0.015)	6.5 (0.054)	0.497 (0.004)	-34.942 (3.907)	12.537 (0.253)	7.837 (0.624)
Supination	Visual mask		131.825 (4.284)	2.994 (6.401)	74.789 (2.809)	4.79 (0.038)	5.75 (0.118)	0.486 (0.005)	59.1 (6.204)	5.143 (1.318)	19.9 (0.918)
	Typical flight		157.764 (4.277)	10.934 (1.687)	72.624 (2.25)	4.834 (0.036)	5.877 (0.116)	0.488 (0.004)	45.432 (5.45)	2.712 (0.813)	17.384 (0.766)
	Submaximum load lifting		174.845 (4.724)	19.056 (2.596)	73.763 (2.575)	4.849 (0.022)	5.853 (0.074)	0.488 (0.004)	40.246 (6.357)	2.236 (0.787)	15.943 (0.804)
Mid-upstroke	Visual mask		87.858 (5.082)	0.208 (6.431)	56.704 (4.135)	4.709 (0.055)	5.474 (0.133)	0.479 (0.004)	68.806 (5.333)	4.228 (1.004)	14.469 (0.673)
	Typical flight		90.357 (5.863)	2.302 (2.41)	40.584 (2.397)	4.743 (0.046)	5.48 (0.116)	0.479 (0.003)	74.342 (3.72)	4.755 (0.735)	12.845 (0.449)
	Submaximum load lifting		95.914 (8.45)	5.794 (2.489)	33.795 (2.888)	4.755 (0.021)	5.455 (0.053)	0.479 (0.003)	76.891 (1.396)	4.622 (0.365)	12.438 (0.655)

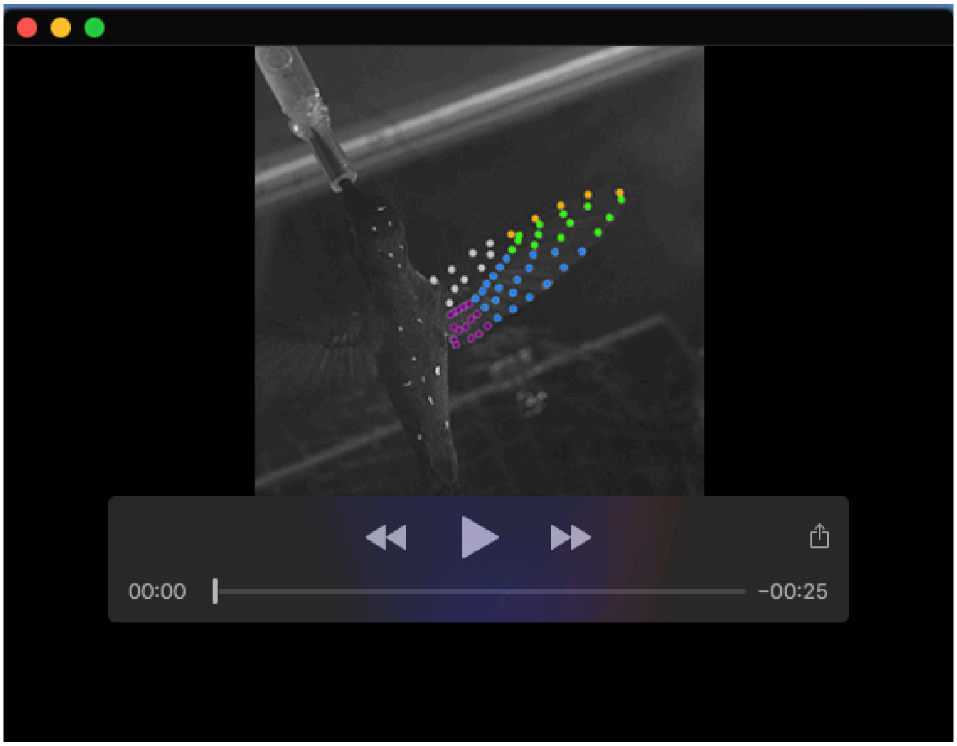

Movie 1. Example wing configurations during typical hoverfeeding, while hoverfeeding in front of a visual mask, and while hoverfeeding together with submaximum load lifting. Digitised landmarks are colour coded as in Figure 3.

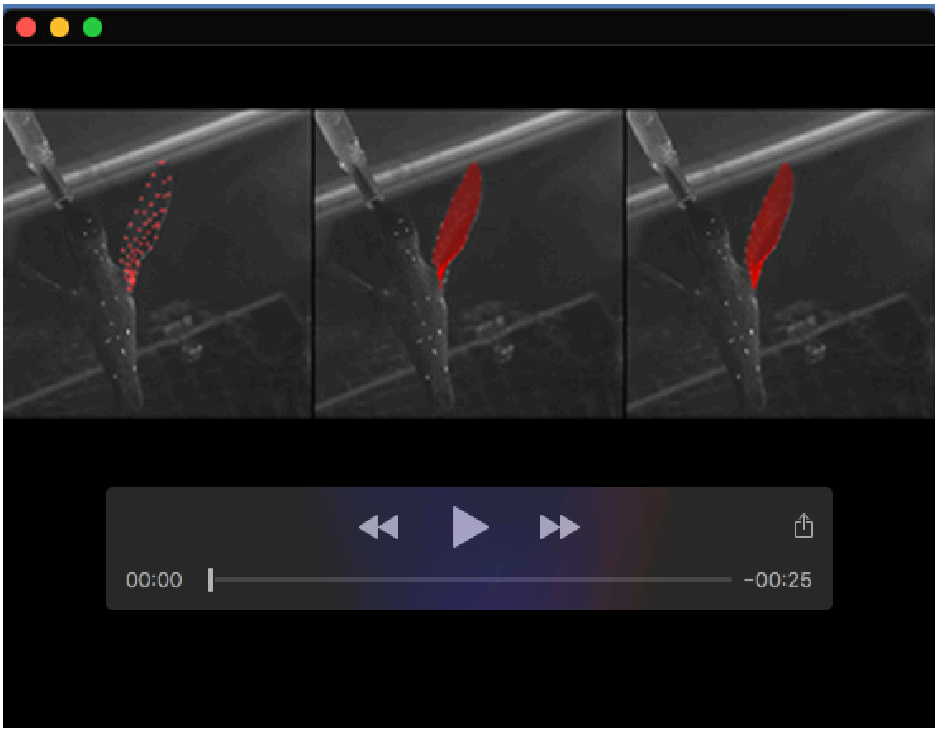

Movie 2. Example wing reconstructions during typical hoverfeeding, while hoverfeeding in front of a visual mask, and while hoverfeeding together with submaximum load lifting. *Left* Digitised landmarks. *Center* Wing surface reconstructed by joining points along the leading and trailing edges. *Right* Wing surface reconstructed by fitting through interior points on the wing.

Journal of Experimental Biology • Supplementary information
